# Supplementary material for: Affective Attitudes in the Face of the COVID-19 Pandemic: The Dynamics of Negative Emotions and a Sense of Threat in Poles in the First Wave of the Pandemic
Source: Int J Environ Res Public Health. 2022 Oct 19;19(20):13497. doi: 10.3390/ijerph192013497 (PMC9642547; doi:10.3390/ijerph192013497)
Supplement: Supplementary file 1 [file ijerph-19-13497-s001.zip › ijerph-1938925-supplementary.pdf]

Supplementary

The exact questions used in the study.

English translation

1. Assess to what extent emotions listed below cause people to experience a sense of threat in the current situation:

Suffering

To a small extent To a high extent

1———————————————————100 (7 in stages 2, 3 and 4)

Helplessness

To a small extent To a high extent

1———————————————————100

Frustration

To a small extent To a high extent

1———————————————————100

Breakdown

To a small extent To a high extent

1———————————————————100

Terror

To a small extent To a high extent

1———————————————————100

Bitterness

To a small extent To a high extent

1———————————————————100

Aversion

To a small extent To a high extent

1————————————————————————100

Disgust

To a small extent To a high extent

1————————————————————————100

Abhorrence

To a small extent To a high extent

1————————————————————————100

Repulsion

To a small extent To a high extent

1————————————————————————100

Humiliation

To a small extent To a high extent

1————————————————————————100

Shame

To a small extent To a high extent

1————————————————————————100

Embarassment

To a small extent To a high extent

1————————————————————————100

Disappointment

To a small extent To a high extent

1————————————————————————————100

Disillusionment

To a small extent   To a high extent

1————————————————————————————100

Sadness

To a small extent   To a high extent

1————————————————————————————100

Sorrow

To a small extent   To a high extent

1————————————————————————————100

Depression

To a small extent   To a high extent

1————————————————————————————100

Envy

To a small extent   To a high extent

1————————————————————————————100

Disdain

To a small extent   To a high extent

1————————————————————————————100

2. To what extent do you feel threatened by the epidemic situation in Poland?

To a small extent   To a high extent

1————2————3————4————5————6————7

To what extent do you feel threatened by the epidemic situation in Europe?

To a small extent — To a high extent

1————2————3————4————5————6————7

To what extent do you feel threatened by the epidemic situation in the whole world?

To a small extent — To a high extent

1————2————3————4————5————6————7

3. Have you contracted the COVID-19 disease?

Stage 1: YES/NO

Stages 2, 3 and 4:

- yes, confirmed with a test
- probably yes, but I haven't had a test
- yes I have, which was confirmed with a test, I have already recovered
- probably yes, but I haven't had a test, I have already recovered
- I haven't contracted the COVID-19 disease
- I don't know

Has someone you know contracted the COVID-19 disease? (Stage 1 only)

YES/NO

Please indicate your sex:

- Woman
- Man
- Other (Stage 1 only)

Please indicate your age (in years):

…

Where do you live? Please indicate the size of the place where you live:

- Village
- Small town (up to 20 000 inhabitants)
- Large town (from 20 000 to 99 000. inhabitants)
- City (from 100 000 to 500 000 inhabitants)
- Large city (more than 500 000 inhabitants)

What is the highest grade or level of school you have completed or the highest degree you have received:

- Primary/middle school
- Vocational
- Secondary education
- Post-secondary education
- Bachelor degree
- Master degree

Are you working a full time job?

YES/NO

In the face of the pandemic, do you have your monthly pay guaranteed?

YES/NO

Original Polish Version

Zaznacz, na ile podane niżej uczucia sprawiają, że ludzie silniej odczuwają zagrożenie w obecnej sytuacji:

Cierpienie

W niewielkim stopniu W znaczącym stopniu

1——————————————————————100 (7 in stages 2, 3 and 4)

Bezradność

W niewielkim stopniu W znaczącym stopniu

1——————————————————————100

Frustracja

W niewielkim stopniu W znaczącym stopniu

1——————————————————————100

Załamanie

W niewielkim stopniu W znaczącym stopniu

1——————————————————————100

Przerażenie

W niewielkim stopniu W znaczącym stopniu

1——————————————————————100

Gorycz

W niewielkim stopniu W znaczącym stopniu

1——————————————————————100

Awersja

W niewielkim stopniu W znaczącym stopniu

1——————————————————————————————100

Obrzydzenie

W niewielkim stopniu W znaczącym stopniu

1——————————————————————————————100

Odraza

W niewielkim stopniu W znaczącym stopniu

1——————————————————————————————100

Wstręt

W niewielkim stopniu W znaczącym stopniu

1——————————————————————————————100

Kompromitacja

W niewielkim stopniu W znaczącym stopniu

1——————————————————————————————100

Wstyd

W niewielkim stopniu W znaczącym stopniu

1——————————————————————————————100

Zażenowanie

W niewielkim stopniu W znaczącym stopniu

1——————————————————————————————100

Zawiedzenie

W niewielkim stopniu W znaczącym stopniu

1——————————————————————————————100

Rozczarowanie

W niewielkim stopniu W znaczącym stopniu

1——————————————————————————100

Zasmucenie

W niewielkim stopniu W znaczącym stopniu

1——————————————————————————100

Przygnębienie

W niewielkim stopniu W znaczącym stopniu

1——————————————————————————100

Depresja

W niewielkim stopniu W znaczącym stopniu

1——————————————————————————100

Zawiść

W niewielkim stopniu W znaczącym stopniu

1——————————————————————————100

Pogarda

W niewielkim stopniu W znaczącym stopniu

1——————————————————————————100

2. W jakim stopniu czujesz się ogólnie zagrożon{y/a} z powodu sytuacji zagrożenia epidemicznego w Polsce?

W niewielkim stopniu W znaczącym stopniu

1————2————3————4————5————6————7

Na ile czujesz się zaniepokojon{y/a} z powodu sytuacji związanej z Coronawirusem na świecie?

W niewielkim stopniu W znaczącym stopniu

1————2————3————4————5————6————7

W jakim stopniu czujesz się zaniepokojon{y/a} z powodu sytuacji związanej z Coronawirusem w Europie?

W niewielkim stopniu W znaczącym stopniu

1————2————3————4————5————6————7

3. Czy jest lub był{/a} Pan{/i} zakażona koronawirusem?

Stage 1: TAK/NIE

Stages 2, 3 and 4:

- Jestem obecnie zakażon{y/a} koronawirusem i test to potwierdził.
- Jest wielce prawdopodobne, że obecnie jestem zakażon{y/a} koronawirusem, ale nie miałam testu
- Był{em/am} zakażon{y/a} koronawirusem i test to potwierdził, ale już wyzdrowiał{em/am}.
- Jest wielce prawdopodobne, że był{em/am} zakażon{y/a} koronawirusem, ale nie miał{em/am}testu
- Nie, nie był{em/am} i nie jestem zakażon{y/a} koronawirusem.
- Nie wiem.

Czy ktoś, kogo Pan{/i} zna jest lub był{/a} zakażona koronawirusem?? (Stage 1 only)

TAK/NIE

Proszę zaznaczyć swoją płeć:

- Kobieta

- Mężczyzna
- Inna (Stage 1 only)

Wpisz swój wiek (w latach):

…

W jakiej miejscowości Pan{/i} mieszka?:

- Wieś
- Małe miasto (do 20 tys. mieszkańców)
- Średnie miasto (od 20 do 99 tys. mieszkańców)
- Duże miasto (od 100 do 500 tys. mieszkańców)
- Wielkie miasto (powyżej 500 tys. mieszkańców)

Jakie jest Pan{a/i} obecne wykształcenie (ostatnio ukończona szkoła)?:

- Podstawowe/ gimnazjalne
- Zasadnicze
- Średnie
- Pomaturalne/ policealne
- Wyższe - licencjat
- Wyższe – magister/inżynier

Czy pracuje Pan{/i} na etacie?

TAK/NIE

Czy w obliczu pandemii ma Pan{/i} zapewnioną ciągłość wynagrodzenia?

TAK/NIE
